# Relationship between Charlson comorbidity index, early recovery and 2-year mortality in elderly patients undergoing surgical treatment of inter-trochanteric fractures: a retrospective analysis

**DOI:** 10.1038/s41598-021-96765-y

**Published:** 2021-08-25

**Authors:** Xu Jianda, Yasuhiro Homma, Yuta Jinnai, Tomonori Baba, Xu Zhuang, Taiji Watari, Sammy Banno, Kazuo Kaneko

**Affiliations:** 1 Department of Orthopaedics, Changzhou Traditional Chinese Medical Hospital, Affiliated to Nanjing University of Traditional Chinese Medicine, 25 North Heping Road, Changzhou, 213000 Jiangsu Province China; 2 Department of Orthopaedic Surgery, Juntendo University, 2-1-1 Hongo, Bunkyo-ku, Tokyo, Japan

**Keywords:** Outcomes research, Translational research

## Abstract

The aim of this study was to evaluate how the Charlson Comorbidity Index (CCI) scores contribute to early recovery and 2-year mortality in elderly patients undergoing surgical treatment of inter-trochanteric fractures. 60 cases with unilateral intertrochanteric fracture were retrospectively analyzed and divided into Low-CCI group (CCI: 1–4) or high-CCI groups (CCI: 5–6). All the patients’ electronic hospital records were reviewed. The preoperative situations (demographic data, comorbidities and fracture conditions), perioperative situations (wait time, operation time, implant choice, blood loss, transfusion or not) and postoperative situations (complications, first time out of bed, function about 1-/2- week and 2-year mortality) were recorded. 51.67% were in low-CCI group and 48.33% in high-CCI group. The survival rates in low- and high-CCI group were 93.5% and 86.2% respectively. According to the functional results of 1- or 2- week after operation, no significant difference was found (P = 0.955, 0.140). Log-rank analysis showed that the main prognostic factors were blood loss, first time out of bed and complication (P < 0.05). Multivariate analysis confirmed that complication and first time out of bed were significant factor on survival rate (P = 0.029, 0.010). Charlson comorbidity index maybe not the indicator of 2-year mortality in older patients with intertrochanteric fractures. In order to improve the prognosis, more attentions should be paid to reduce the complications and encourage postoperative earlier excise out of bed.

## Introduction

As a common fracture in elderly people, the intertrochanteric fracture comprises about 8–10% of all fractures^1^. Although fracture is not the direct cause of death, it can induce a progressive deterioration of physical condition and increase the mortality. The mortality of intertrochanteric fractures displays a rising trend due to increased life expectancy and osteoporosis^2^.

Surgery usually supplies an earlier mobilization to reduce complications and mortality, and improves the independent living ability in older patients^3^. To now, surgery at the earliest opportunity has being the main choice for intertrochanteric fractures, but the relative higher mortality is still a major concern to our clinicians. The trauma reaction, physiological decline and underlying diseases are still the important issues requiring our emphasizing^4^. Considering the difficulty and complexity, understanding the risk factors of mortality is very important for supplying safer and better treatment.

Charlson comorbidity index (CCI) is a system assigned to weigh the morbidity, which is used to assess the probability of survival^5^. Previous researches have confirmed that CCI is an important tool to predict the prognosis (30-, 90-days mortality) of patients with hip fractures^6^. Haentjens et al. used life-table method and found that women and men (older than 80 years old) having a hip fracture often brought them with excess annual mortality at 1, 2, 5 and 10 years after injury^7^. Considering the following deleterious effects of hip fractures, earlier evaluating the risk factors about the mid- to long-term mortality are very important.

The aim of this present study was to evaluate the role of Charlson comorbidity index (CCI) on 2-year mortality in older patients with intertrochanteric fractures. In addition, we investigated other main risk factors influencing the prognosis.

## Materials and methods

This retrospective study was designed and approved by the Ethics Review Committee of Juntendo University. All methods were performed in accordance with the relevant guidelines and regulations. Informed consent was obtained from all patients.

From Jan. 2013 to Dec. 2017, 71 patients with intertrochanteric fractures were retrospectively analyzed. Of them, 11 patients were excluded: 7 patients with conservative treatment, 2 patients with multiple fractures and 2 patients under 65.

60 patients, ASA I-IV, aged 65–100 (average, 81.70 ± 8.13), BMI 14.7–29.42 (average, 21.34 ± 3.45), unilateral intertrochanteric fractures were analyzed finally. Of them, 17 males and 43 females. The preoperative wait time was 2–21 (average, 7.37 ± 3.83) days. The fractures were classified according to AO/ASIF system, in which 25 type A1 fractures, 33 type A3 fractures and 2 type A3 fractures. All the enrolled patients got surgery with intramedullary nail (59 cases) or dynamic hip screw (only 1 case). The CCI score was assessed first and divided the enrolled patients into Low-CCI group (n = 31; CCI: 1–4) and High-CCI group (n = 29; CCI: 5–6).

The inclusion criteria were aged over 65; unilateral intertrochanteric fractures with surgery; no other diseases before affecting the lower limb function; a complete clinical data. Some patients were excluded if they were younger than 65; if they chose conserve therapy; if they had pathological fractures; if they had diseases before affecting the lower limb function; if their clinical data defected.

No significant correlation was found in age, gender, side, AO type, stability, low limber surgery, preoperative wait time, complication, transfusion, implant style, ASA classification, wait days, blood loss, comorbidities, operation time and first time out of bed (P > 0.05, Table 1).

**Table 1:** The baselines of patients from the low- and high-CCI groups (Created by SPSS 24.0 software).

	Low-CCI group	High-CCI group	P
Age	81.61 ± 9.22	81.79 ± 6.94	0.132
Gender(M/F)	11/20	6/23	0.258
BMI	21.45 ± 3.65	21.22 ± 3.27	0.521
Side(L/R)	15/16	18/11	0.312
AO(A1/A2/A3)	13/16/2	12/17/0	0.367
Stability(Y/N)	22/9	17/12	0.418
Low limber surgery(Y/N)	6/25	6/23	0.897
Complication(Y/N)	2/29	1/28	0.594
Transfusion(Y/N)	9/22	11/18	0.586
Implant(Nail/DHS)	31/0	28/1	0.483
ASA(I/II/III/IV)	8/0/17/6	2/1/21/5	0.168
Wait time	7.10 ± 3.80	7.66 ± 2.90	0.569
Blood loss	73.06 ± 61.87	75.34 ± 57.13	0.760
Comorbidity(Y/N)	17/14	16/13	0.979
Operation time	70.55 ± 26.27	70.72 ± 20.0	0.275
First time out of bed	1.33 ± 0.88	1.24 ± 0.69	0.389

*ASA* American Society of Anesthesiologists Classification; Gender(M/F): Gender(Male/Female); Side(L/R): Side(Left/Right); Stability: the fracture is stable or not; Low limber surgery: the patient has low limber surgery before or not; Complication(Y/N): the patient has complication before or not; Transfusion(Y/N): the patient has blood transfusion before or not; Comorbidity(Y/N): the patient has comorbidity or not.

The operations were performed by the same team major in hip. According to the regime of our rehabilitation, the physiotherapist would help patients to mobilize from the second day after operation. The patients attended rehabilitation twice a day. If possible, they would excise out of bed as early as possible (usually the second day after operation). The following rehabilitation projects mainly based on patients’ own physical conditions to accelerate muscle strength, limb coordination and body balance recovery.

### Study clinical parameters

Follow-up was defined from the operation day. 2-year mortality was defined the survival rate of all enrolled patients at 2 year.

The preoperative situations (demographic data and comorbidities), perioperative situations (wait time, operation time, implant choice, blood loss, transfusion or not) and postoperative situations (complications, first time out of bed and 2-year mortality) were got from the patients’ electronic hospital records. And all the data was manual reviewed independently by two different researchers.

### Radiographic evaluation

The fracture classification and stability^8^ were assessed independently by two different radiologists using PACS system.

### Functional evaluation

Functional result at 1-week or 2-week: The function was evaluated by two doctors based on rehabilitation record. The classifications listed as follow: no walking (The patients couldn’t walk even with a walker), walking with walker (The patients could walk with a walker), walking with stick (The patients could walk with a stick) and self-walking (The patients could walk without any help).

### Statistical analysis

SPSS 24.0 (SPSS Inc, Chicago, IL, USA) was employed for all statistical analyses. Kolmogorov–Smirnov test was used to assess the normality distribution of continuous variables. Mean (standard deviation) or median (interquartile range) were used as appreciate. The t-test or χ^2^ test were used to evaluate difference or association between groups. The relationships between the survival and possible risk factors were assessed by univariate log rank test, Cox’s proportional hazards regression model with a forward conditional stepwise procedure was employed to determine the acting factors. Kaplan–Meier curves were plotted to assess the differences in 2-year mortality. The significance level was set at P < 0.05.

## Results

**Figure 1:**
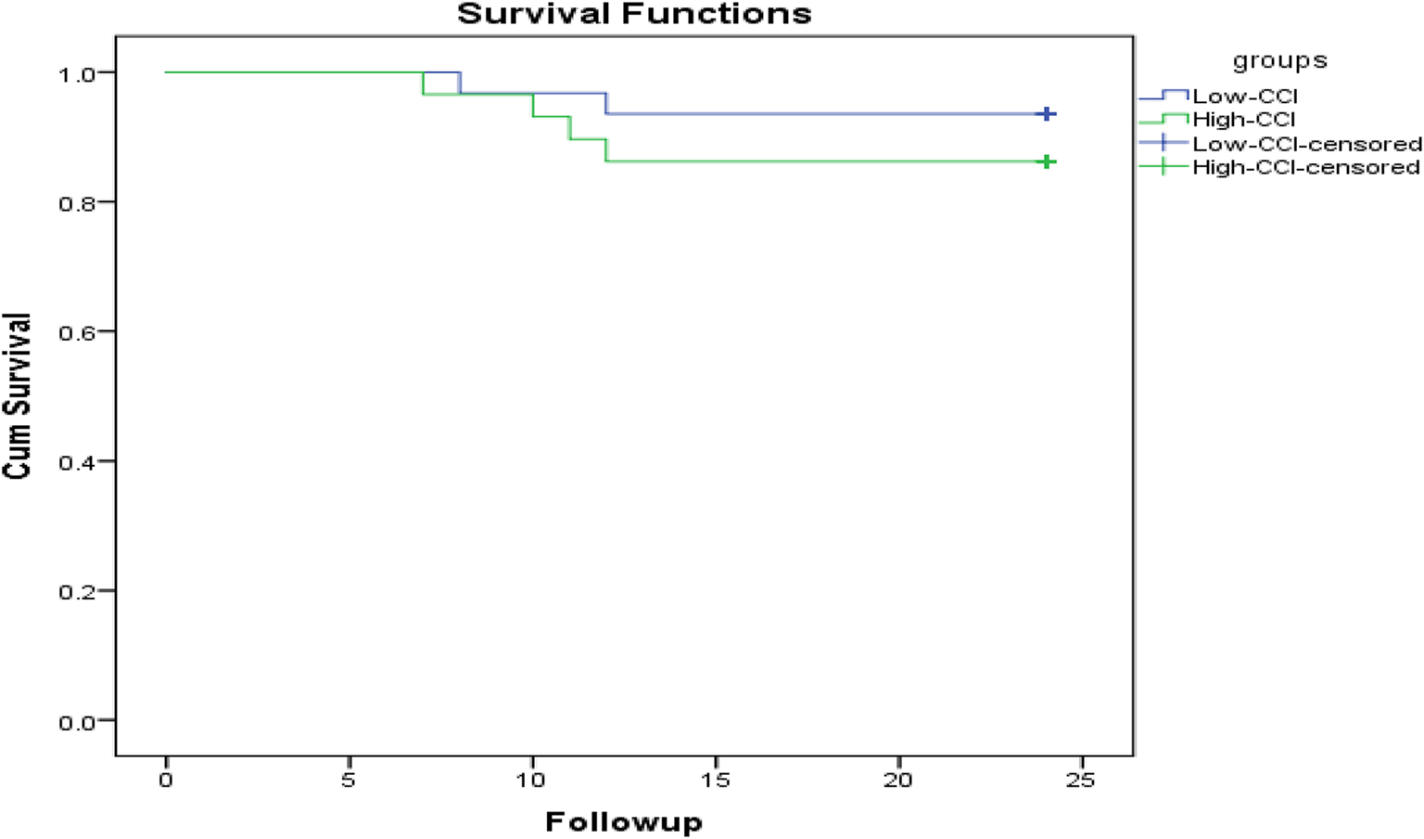
A Kaplan–Meier plots for overall survival for low-CCI group compared to high-CCI group. (Created by SPSS 24.0 software).

All 60 patients got followed-up and bone united uneventfully. Of them, 51.67% were in low-CCI group and 48.33% in high-CCI group. All survival data were available and the survival rates in low- and high-CCI group were 93.5%, 86.2% respectively (Fig. 1). Three complications were found postoperatively. One patient got urinary tract infection and was cured by antibiotic treatment. Two others had pressure sore, and the wound healed after dressing change. No other secondary displacement, superficial or deep infection was found.

### The earlier postoperative functional results of the low- and high-CCI groups

According to the functional results of 1- or 2- week after operation, no significant difference was found between low- and high-CCI groups (P = 0.955, 0.140, Table 2). The first time out of bed postoperatively is 1.29 ± 0.79 days.

**Table 2:** The earlier postoperative functional results of the low- and high-CCI groups (Created by SPSS 24.0 software).

	No walking	Walker	Stick	Self-walking	P
**Functional result of 1 week after operation**					
Low-CCI group	9	19	1	2	0.955
High-CCI group	8	19	1	1	
**Functional result of 2 week after operation**					
Low-CCI group	4	15	5	7	0.140
High-CCI group	1	21	1	6	

At 1 week after operation, only 3 patients (5.0%, Table 2) could walk by themselves, 17 patients (28.3%, Table 2) couldn’t walk, and others needed the help of walker or stick. At 2 weeks after operation, 13 patients (21.7%, Table 2) could walk by themselves, only 5 patients (8.3%, Table 2) couldn’t walk, and others needed the help of walker or stick.

### The prognostic factors and survival postoperatively

Log-rank analysis showed that the main prognostic factors were blood loss, first time out of bed and complication (P < 0.05, Table 3). No significant correlation was found with other factors (P > 0.05, Table 3).

**Table 3:** Univariate analysis of prognostic factors of on 2-year mortality in older patients with intertrochanteric fractures (Created by SPSS 24.0 software).

	Survival rate (%)	P
Age(≥ 80/ < 80)	92.3/85.7	0.429
Gender(M/F)	94.1/90.7	0.879
BMI	85.7/93.8	0.286
ASA(I/II/III/IV)	80.0/100.0/94.7/81.8	0.399
AO (A1/A2/A3)	100.0/81.8/100.0	0.070
Comorbidity (N/Y)	92.6/87.9	0.571
Side (L/R)	90.9/88.9	0.797
Stability	94.9/81.0	0.078
CCI(≥ 5/ < 5)	86.2/93.5	0.343
Limb surgery(N/Y)	91.7/83.3	0.384
Wait time (d)	91.3/89.2	0.763
Operation time(min)	83.3/94.4	0.149
Blood loss (ml)	77.3/97.4	0.011
Transfusion (Y/N)	80.0/95.0	0.063
Implant (Nail/DHS)	89.8/100.0	0,744
First time out of bed (1d/ > 1d)	94.1/66.7	0.013
Complication (N/Y)	93.0/33.3	0.000

For example: “Complication (N/Y) 93.0/33.3”: The Survival rate in patients without complication was 93.0%, while 33.3% in patients with complication.

### Multivariate analysis of prognostic factors in patients with intertrochanteric fractures

Multivariate analysis confirmed that complication and first time out of bed were significant factor on survival rate (P = 0.029, 0.010, Table 4).

**Table 4:** Multivariate analysis of prognostic factors in patients with intertrochanteric fractures (Created by SPSS 24.0 software).

	B	Wald	P	Hazard ratio	95% Confidence Interval	
					Lower	Upper
Complication	2.567	4.784	0.029	13.021	1.306	129.841
First time out of bed	0.849	6.603	0.010	2.337	1.223	4.464

## Discussion

Surgery at the earliest opportunity has being the main choice for intertrochanteric fractures in older patients. However, the relative higher mortality is still a major concern to our clinicians. Schurch et al. proposed a prospective survey of hip fracture and found that the 1-year mortality reached to 23.8%. Only 62.6% patients returned to their previous living after one year, and more care-intensive environment were still needed in 17.9% patients^9^. Kusen et al. implemented a multidisciplinary care pathway to improve the prognosis of older Patients with traumatic hip fractures. And they found that it could significantly reduce the mortality by 2.9% at 30 days, 3.4% at 90 days and 0.1% at 1 year^10^. Understanding and processing the risk factors of mortality in time maybe a prefer choice to reduce postoperative mortality. Our study demonstrated that the overall mortality rate was just 10.0%. Only three complications (1 urinary tract infection and 2 pressure sores) were found postoperatively. No other secondary displacement, superficial or deep infection was found.

### Charlson comorbidity index (CCI)

CCI was a most widely comorbidity measure method used to assess the probability of survival. Previous research had confirmed that CCI was an important tool to predict the prognosis (30-, 90-days mortality) of patients with hip fractures. What’s more, the CCI had been confirmed to predict in-hospital mortality and readmission after orthopaedic surgery accurately^11,12^.

Considering the following deleterious effects of hip fractures, earlier evaluating the risk factors about the mid- to long-term mortality are very important. Lei Jiang et al. retrospective analyzed 1057 hip fracture patients aged above 60 years and found that Charlson comorbidity index was correlated with 5-year mortality after surgery^13^. In proximal humerus fractures, patients with high CCI (≥ 5) had a higher mortality risk 4.6 (95% CI [2.4–9.0]) compared to those with CCI < 5. According to the ROC curve analysis, the previous study confirmed the optimal cutoff value for the CCI was ≥ 3.5 for death after hip fracture surgery^14^. In this present study, we divided the enrolled patients into two groups: Low-CCI group (n = 31; CCI: 1–4) and High-CCI group (n = 29; CCI: 5–6). We analyzed the risk factors about 2-year mortality in older patients with intertrochanteric fractures. But no significant relationship was found between CCI and mortality rate in this present study. Maybe small sample size and age composition were main influence factors. The average age of all enrolled patients was 81.7 ± 8.1 years old, and patients ≥ 80 was about 63.9% (39/61).

Gilbert et al. had got the similar conclusion and thought that high comorbidity index was not related with high morbidity and mortality^15^. But they also pointed out that constrained total hip arthroplasty provided better function to the elderly patients with intertrochanteric fractures. Palanisamy et al. compared the functional outcomes in geriatric hip fractures between different implants. The final results showed that in patients with high CCI, THR usually provided faster recover^16^. Whether CCI combined with other factors can bring better clinical significance? It remains to be further studied in future.

### Complications

Compared with intra-capsular fracture, patients with intertrochanteric fractures usually accompanied with increased blood loss^17^. It’s a potential reason for hypotension and hemodynamic disturbance during perioperative period. These fluctuations predisposed the higher complication rate. Chen et al. analyzed the risk factors for 1-year mortality in patients with acute hip surgery and strongly recommended that controlling and co-caring postoperative complications could significantly reduce the mortality^18^.

In our present study, multivariate analysis confirmed that complication was a significant factor on survival rate. Other studies had also got the similar conclusions. Zhao et al. performed a multivariate analysis and found that BNP, APACHE II score and complications after fracture were reference indexes for predicting perioperative mortality in elderly patients with intertrochanteric fracture^19^. Therefore, it is very important to control complications during perioperative period.

### First-time out of bed

Earlier mobilization has been regarded as an important factor for better function after lower limb surgery. It can reduce complications and improve the independent living ability in older patients^20^. In present study, the mean first time out of bed postoperatively was 1.29 ± 0.79 days under the guidance of physiotherapist. The earlier weight-bearing time was much shorter than others’ reports. Duymus et al compared the result of intra- and extramedullary implants in treatment of intertrochanteric fractures and found that proximal femoral nail had better clinical results. The full weight-bearing time was significantly reduced to 1.25 ± 0.40 months^21^.

Our multivariate analysis confirmed that first time out of bed was a significant factor on survival rate. And we thought earlier weight-bearing is a better method to reduce the rate of complications and mortalities. Some surgeons worried that earlier weight bearing would bring a higher implant failure rate, but our follow-up study didn’t lead to a higher failure rate. In our hospital, the physiotherapists usually help patients weight bearing from the second day after operation if permitted. And the rehabilitation content was adjusted simply according to the actual situation of each patient. Early mobilization can accelerate muscle strength, limb coordination and body balance recovery. Of course, the lower BMI in Japan may have some impact on higher failure rate. To now, there is no sufficient evidence to determine the better weight bearing time. Further studies are needed to be conducted to express it and related risk factors.

At present, there is another view that arthroplasty was a prefer choice for older patients with intertrochanteric fractures. It provided an earlier weight bearing and better functional prognosis^22^. However, there is still controversial about arthroplasty or internal fixation^23^.

### Limitations

Some limitations could be found in this study. First, it’s a retrospective study with small sample size. The small size reduced the power but provided our own real experience. Second, as a large private hospital, frequently-used hip function scoring standards were not regularly registered. And we used our own semi-quantitative based on the patients’ electronic hospital records. Third, due to Japanese medical system, not all patients were died in our hospital. The concrete causes of death were still uncertain Fourth, this retrospective study just came from our own hospital, and multicenter and more sample studies are necessary in future.

## Conclusion

Therefore, Charlson comorbidity index maybe not the indicator of 2-year mortality in older patients with intertrochanteric fractures. In order to improve the prognosis, more attentions should be paid to reduce the complications and encourage postoperative earlier excise out of bed.

## Data Availability

All data generated or analyzed during this study are included in this published article and are available from the corresponding author on reasonable request.
